# Photocatalytic activities of coke carbon/g-C_3_N_4_ and Bi metal/Bi mixed oxides/g-C_3_N_4_ nanohybrids for the degradation of pollutants in wastewater

**DOI:** 10.1080/14686996.2016.1235962

**Published:** 2016-10-12

**Authors:** Marta Sierra, Emma Borges, Pedro Esparza, Jorge Méndez-Ramos, Jesús Martín-Gil, Pablo Martín-Ramos

**Affiliations:** ^a^ Chemical Engineering Department, University of La Laguna, Santa Cruz de Tenerife, Spain; ^b^ Chemical Department, University of La Laguna, Santa Cruz de Tenerife, Spain; ^c^ Physics Department, University of La Laguna, Santa Cruz de Tenerife, Spain; ^d^ Advanced Materials Laboratory, ETSIIAA, University of Valladolid, Palencia, Spain; ^e^ EPSH, University of Zaragoza, Huesca, Spain

**Keywords:** Carbon nitride, coke, bismuth mixed oxides, photocatalysis, wastewater pollutants, 60 New topics/Others, 104 Carbon and related materials, 205 Catalyst / Photocatalyst / Photosynthesis

## Abstract

Different g-C_3_N_4_ composite systems (coke carbon/g-C_3_N_4_, Bi/Bi_2_WO_6_/g-C_3_N_4_ and Bi/Bi_2_MoO_6_/g-C_3_N_4_) have been assessed as photocatalysts for wastewater pollutants removal. The coke carbon/g-C_3_N_4_ hybrid, produced by thermal treatment at 550 °C of a composite made from melamine cyanurate and coke, only showed activity under UV-light irradiation. On the other hand, inorganic Bi spheres/Bi mixed oxides/g-C_3_N_4_ nanohybrids (Bi/Bi_2_WO_6_/g-C_3_N_4_ and Bi/Bi_2_MoO_6_/g-C_3_N_4_ composites), produced by thermal reduction of Bi_2_WO_6_ or Bi_2_MoO_6_ by g-C_3_N_4_, exhibited a remarkable red-shift, up to 620 nm, and allowed the visible-light driven degradation of the contaminant, albeit in combination with some adsorption.

## Graphical abstract

**Figure:**
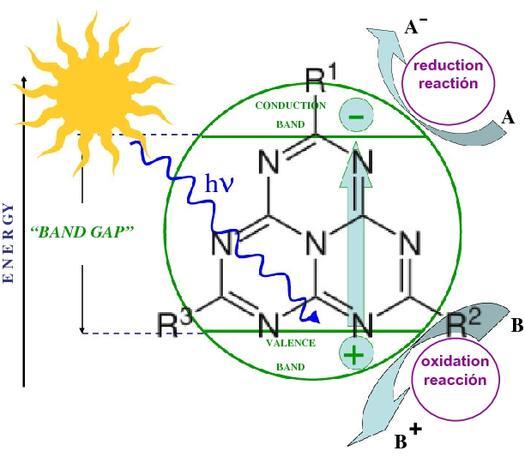

## 1. Introduction

Population growth, improvements in living standards and the increasing pollution of natural resources are major contributors to environmental problems. From an environmental viewpoint, advanced oxidation processes (AOPs) are one of the most sustainable ways of removing pollutants present in both aqueous and gaseous effluents.[1,2] Amongst these, the field of heterogeneous photocatalysis stands out as the one in which technology has undergone the most significant development over the last four decades, owing to its versatility and low cost.[3]

The heterogeneous photocatalytic process has been deemed as particularly promising for the removal of certain persistent pollutants that cannot be removed by conventional wastewater treatments.[4–8] Several semiconductor photocatalysts have been widely studied in the past decades, mainly TiO_2_ and ZnO.[9–11] Based on the band gap usually shown by TiO_2_ and ZnO compounds, their photocatalytic activity requires ultraviolet light (*λ* < 400 nm), which accounts for only *c*. 4% of the global solar radiation. On the other hand, the visible range represents around 42% of aforementioned global solar radiation, which encourages the development of visible light-driven photocatalysts, such as carbon nitride (C_3_N_4_). This material has lately been in the spotlight due to its easily tunable band gap, chemical inertness and stability. Although there are several allotropes of carbon nitride, graphitic carbon nitride (g-C_3_N_4_) has been shown to be the most stable under ambient conditions.[12–17] The reactivity of this polymeric semiconductor, mainly composed of carbon and nitrogen,[18] can be tuned without major changes in its overall composition. It has found application in energy conversion,[19,20] hydrogen and carbon dioxide storage,[21–24] gas sensors,[25,26] solar cells,[15,27,28] water splitting [29–32] and organic pollutants degradation.[14,16,17,33]

Nevertheless, in spite its moderate band gap (~2.7 eV), this polymer tends to exhibit high recombination rates of electron–hole pairs, thus limiting its practical applications. In order to solve these restrictions and to improve its photocatalytic properties, several strategies have been reported, such as the design of heterojunction composites [34] or the modification of the g-C_3_N_4_ preparation.[35] The first approach, based on the separation of the electron–hole pairs, is especially suitable to improve the quantum efficiency and photocatalytic performance.

For combination purposes with g-C_3_N_4_, as an alternative to TiO_2_ and ZnO catalysts, metal oxides where the metal has a complete *d* shell (e.g. Bi_2_O_3_, In_2_O_3_ or Ga_2_O_3_) and complex metal oxides containing cations of *d* ^0^ and/or *d* ^10^ electronic configurations (i.e. niobates, vanadates, tungstates, titanates, tantalates and germanates) have been reported as very successful photocatalysts.[36–40] These metal oxides or complex metal oxides possess steep absorption edges in the visible-light region, different from the more structured spectrum of TiO_2_-doped materials.

In this paper, different g-C_3_N_4_ composite systems (coke carbon/g-C_3_N_4_, Bi_2_WO_6_/g-C_3_N_4_ and Bi_2_MoO_6_/g-C_3_N_4_) have been assessed in order to enhance g-C_3_N_4_ intrinsic photocatalytic properties in the degradation of methylene blue (MB), as a wastewater pollutant molecule model. This study aims to build on research previously conducted on the graphene/g-C_3_N_4_ system by Sierra et al*.* [41] and Li et al*.* [42] and on the research effort undertaken by other authors on the Bi-W(Mo) mixed oxides/g-C_3_N_4_ system, such as Tian et al*.* [43] on g-C_3_N_4_/Bi_2_WO_6_; Xiong et al*.* [44] on g-C_3_N_4_/β-Bi_2_O_3_; Ohno et al*.* [45] and Aslam et al*.* [46] on g-C_3_N_4_/WO_3_; Dong et al*.* [47] on organic Bi-spheres/C_3_N_4_; and Ma et al*.* [48] on a g-C_3_N_4_/RGO/Bi_2_WO_6_ catalyst with intermediate composition between the two studied systems.

## 2. Experimental section

### 2.1. Materials synthesis

Melamine cyanurate (CAS No. 37640-57-6) was supplied by Nachmann S.r.l. (Milan, Italy) with purity higher than 99%; potassium tungstate (CAS No. 7790-60-5, 94%), potassium molybdate (CAS No. 13446-49-6, 98%), bismuth nitrate (CAS No. 10035-06-0, 98%), potassium methoxide (CAS No. 865-33-8, 95%), sulfuric acid (CAS No. 7664-93-9, ACS reagent), sulfolane (CAS No. 126-33-0, 99%) and methylene blue (CAS No. 122965-43-9, dye content ≥82%, certified by the Biological Stain Commission) were purchased from Sigma-Aldrich Quimica SL (Madrid, Spain). All reagents were used without further puriﬁcation.

g-C_3_N_4_ was synthesized through direct heating of sulfuric acid-treated melamine cyanurate (SATS) –the product of a catalytic and disrupting reaction of a melamine and cyanuric acid adduct with H_2_SO_4_ 1 M– according to the procedure reported by Dante et al*.* [49]. Five grams of SATS powder were heated to 550 °C (at a heating rate of 10 °C min^−1^) for 50 min in a sealed Vycor^®^ glass vial using a convective tubular oven (Carbolite GVA 12/900; power: 5.460 kW, heating length: 900 mm, *T*_max_: 1200 °C) under nitrogen flow and then cooled at a rate of 10 °C min^−1^, giving a light-yellow product which consists of dehydrated melamine cyanurate, according to the reaction in [50]:

$${\text{C}}_{\text{3}}{\text{N}}_{\text{3}}{\left({\text{NH}}_{\text{2}}\right)}_{\text{3}}{\;\text{+ C}}_{\text{3}}{\text{N}}_{\text{3}}{\left(\text{OH}\right)}_{\text{3}}\to {\;\text{C}}_{\text{6}}{\text{N}}_{\text{7}}\left(\text{NH}\right)\left({\text{NH}}_{\text{2}}\right){\;\text{+ 3H}}_{\text{2}}\text{O}$$

A thorough characterization of the resulting material can be found in previous works.[49–51] The choice of 550 °C for the thermal treatment is supported by the superior photocatalytic performance of the g-C_3_N_4_ resulting polymerization/condensation reaction at this temperature.[32]

For the preparation of S1 and S2 samples, a mixture consisting of 10% graphitizable carbochemical coke (C_coke_), supplied by ArcelorMittal, and 90% SATS was dispersed in water with 10% sulfolane and, after sonication for 10 min, it was heated under reflux with vigorous stirring at 150 °C for 48 h. The resulting product, washed with water and alcohol and then dried at 60 °C overnight, basically consisted in a composite of C_coke_ and melamine cyanurate (S1). Heating of S1 at 550 °C for 50 min yielded composite S2, a mixture of C_coke_ and polymeric dehydrogenated carbon nitride (g-C_3_N_4_).

The Bi/Bi_2_WO_6_/C_3_N_4_ composite was prepared by dissolving 0.33 g of potassium tungstate dihydrate (K_2_WO_4_·2H_2_O) and 0.97 g of bismuth nitrate pentahydrate (Bi(NO_3_)_3_·5H_2_O) in 60 ml of 10% potassium methoxide, followed by ultra-sonication for 30 min. The resulting precipitate was centrifuged and washed repeatedly with deionized water and alcohol and then dried at 60 °C overnight. Subsequently, it was mixed in an agate mortar with 0.1 g of g-C_3_N_4_ and heated at 550 °C for 50 min under nitrogen flow. The composite that resulted (S3) was a slightly greenish-yellow product.

The preparation of the Bi/Bi_2_MoO_6_/C_3_N_4_ composite (S4) was entirely analogous to that of Bi/Bi_2_WO_6_/C_3_N_4_, but using K_2_MoO_4_. This composite showed two phases with greenish-yellow and metallic colors. The specific surface areas of S1, S2, S3, S4 samples were 4.8, 45.8, 1.5, 1.3 m^2^ g^–1^, respectively.

### 2.2. Materials characterization

Infrared spectra were recorded with a Thermo Scientific (Waltham, MA, USA) Nicolet iS50 Fourier transform infrared (FTIR) spectrometer, equipped with a built-in diamond attenuated total reflectance (ATR) system, in order to identify the chemical functional groups.

X-ray powder diffractograms of the samples were obtained using a Bruker (Billerica, MA, USA) D8 Advance Bragg-Brentano diffractometer, in reflection geometry.

Scanning electron microscopy (SEM) and transmission electron microscopy (TEM) images were collected with an FEI (Hillsboro, OR, USA) Quanta 200FEG microscope equipped with a Genesis energy-dispersive X-ray (EDS) spectrometer system and with a JEOL (Akishima, Tokyo, Japan) JEM-FS2200 HRP microscope equipped with an Oxford instruments INCA Energy TEM 250 EDS probe, respectively.

The diffuse reflectance spectra of the samples were obtained by means of a UV-visible Agilent (Santa Clara, CA, USA) Cary 3 spectrometer equipped with an integration sphere. The materials were not diluted in any matrix to avoid a decrease in the absorbance. The spectra were recorded in diffuse reflectance mode and transformed by the instrument software to equivalent absorption Kubelka-Munk units.

### 2.3. Photocatalytic activity evaluation

The photocatalytic activity of the different g-C_3_N_4_ based composites was evaluated using methylene blue as a wastewater pollutant model under artificial light. Photo-oxidation experiments were carried out using a 150 W visible Hamamatsu (Hamamatsu City, Shizuoka, Japan) L2274 Xe-lamp and a 150 W Heraeus (Hanau, Germany) TQ-150 UV Hg-lamp. The reaction was performed in a stirred photo-reactor with a capacity of 250 ml, placing the UV and visible lamps inside the reactor and keeping the temperature constant at 20 °C. A more detailed description of the experimental setup used for the photodegradation assays can be found in [52].

In all experiments, the initial concentrations of the pollutant (MB) and the photocatalyst were 50 mg l^−1^ and 0.5 g l^−1^, respectively. Both materials were placed into the reactor under continuous stirring and aerated by a pump, so as to provide oxygen, for 240 min. Aliquots of wastewater were taken at different times during the photoreaction in order to evaluate the photocatalytic activity of the materials under study. The pollutant degradation was determined by analyzing the MB concentration using an Agilent (Santa Clara, CA, USA) Cary 50 UV-vis spectrophotometer. The contribution of the adsorption of the pollutant on the photocatalytic surface was evaluated in the same reaction conditions, but in the dark.

## 3. Results and discussion

### 3.1. Materials characterization

#### 3.1.1. X-ray powder diffraction analysis

**Figure 1.**
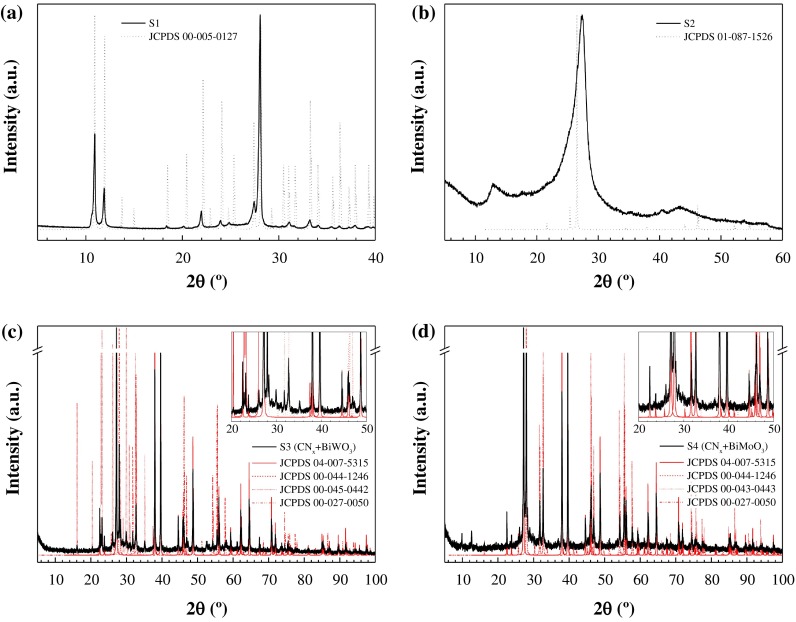
X-ray powder diffraction patterns of: (a) C_coke_/melamine cyanurate (S1), (b) C_coke_/g-C_3_N_4_ (S2), (c) Bi/Bi_2_WO_6_/g-C_3_N_4_ (S3), and (d) Bi/Bi_2_MoO_6_/g-C_3_N_4_ (S4) composites. JCPDS patterns have also been included for comparison purposes.

As depicted in Figure 1(a), the diffractogram of sample S1 (solid black line) shows a good agreement with that of melamine cyanurate (dotted black line): the peaks appear at the expected theta angles, and differences in intensity can be ascribed to the Bragg–Brentano geometry of the instrument used. On the other hand, the XRD pattern of the sample condensed at 550 °C (S2, Figure 1(b)) provides direct evidence of the formation of g-C_3_N_4_, since it shows two distinct diffraction peaks around 27.4° (intense) and 12.8° (weak), corresponding to the (0 0 2) reflection with interplanar distance of 3.25 Å and to the (1 0 0) diffraction of in-plane structural periods of tri-s-triazine unit (*d* = 6.91 Å), respectively.[53,54] The shift in the first peak versus that in JCPDS 87-1526 pattern is related to differences in the treatment temperature (a higher treatment temperature induces a tightening of the interplanar distance and stronger interplanar interaction).[50]

The powder diffraction patterns of S3 (see Figure 1(c)) and S4 (Figure 1(d)) show the presence of Bi metal (JCPDS 44-1246), bismuth oxide (JCPDS 27-0050) and either bismuth tungstate (JCPDS 45-0442) or bismuth molybdate (JCPDS 43-0443), according to the ICDD database. The peak at *c*. 2*θ* = 28° may be ascribed – as noted above – to (0 0 2) in g-C_3_N_4_, but it can also be assigned to (1 1 3) diffraction plane of Bi_2_MO_6_ (M = W, Mo). Peaks at 2*θ* = 38°, 40°, 56°, 62.5°, 65° and 72° can be attributed to (1 0 4), (1 1 0), (0 2 4), (1 1 6), (1 2 2) and (2 1 4) diffraction planes in Bi-nanospheres (JCPDS 04-007-5315). Peaks at 2*θ* = 33°, 48°, 57° and 59° can be attributed to the (2 0 0), (2 2 0), (3 1 3) and (2 2 6) diffraction planes of Bi_2_MO_6_ (M = W, Mo).

#### 3.1.2. TEM and SEM images

**Figure 2.**
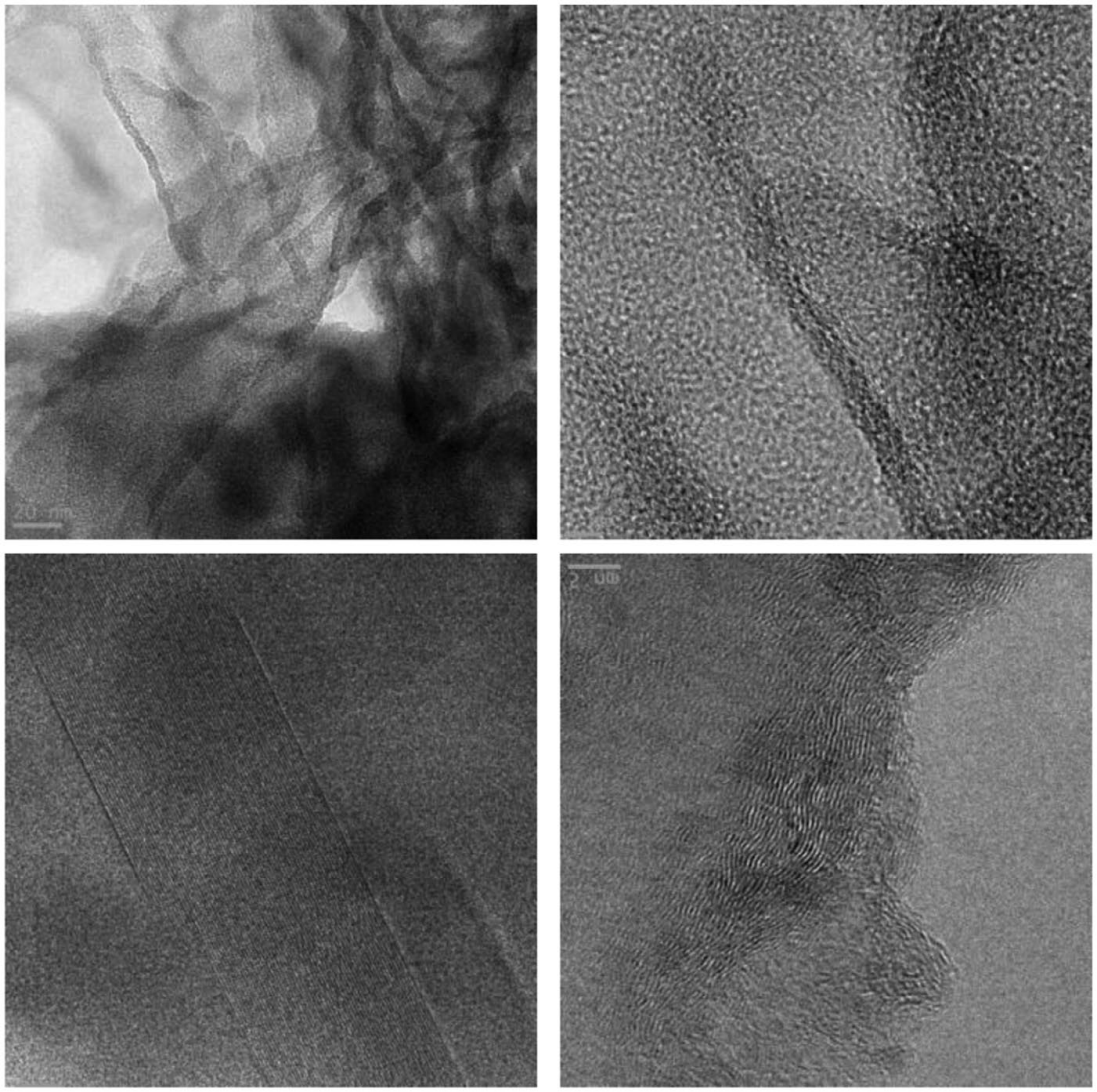
TEM micrographs of S2 (C_coke_/g-C_3_N_4_) composite.

The texture of the C_coke_/g-C_3_N_4_ sample (S2) was studied by TEM (Figure 2). The micrographs evidence the graphenic structure of the composite and provide details on flakes, mosaic structures and domains. It can be observed that the nanosheets of polymeric carbon nitride tend to form crumpled surfaces. These conformations seem to originate from the curled surfaces, which tend to roll up and then shrink by effect of the stabilization needed by the particle sheets, which were constituted by several layers.[50]

**Figure 3.**
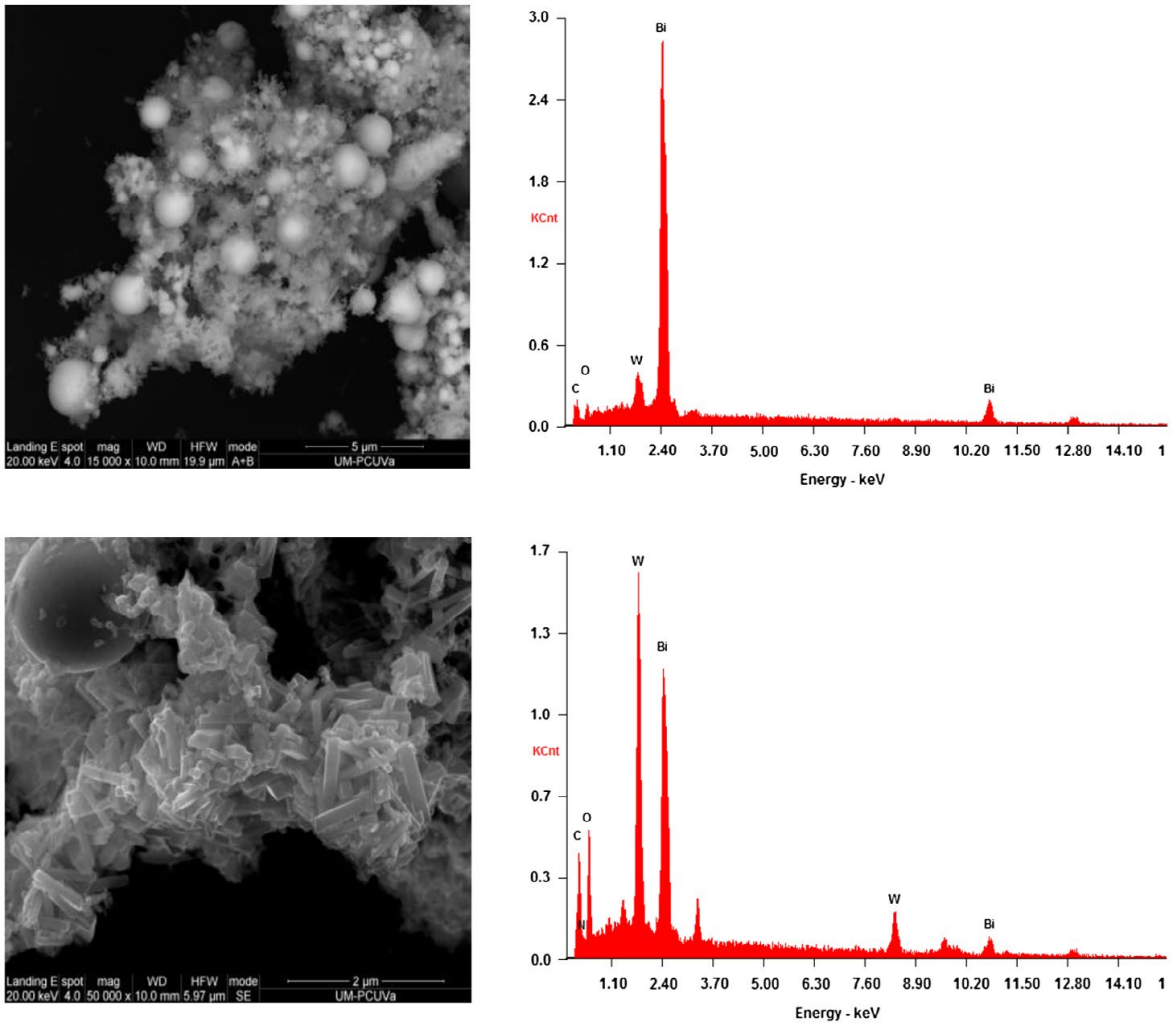
SEM micrographs (left) and EDS analyses (right) of S3 (Bi/Bi_2_WO_6_/g-C_3_N_4_) composite. The data on top are for the bulk, while the data on the bottom correspond to the Bi_2_WO_6_/g-C_3_N_4_ matrix.

**Figure 4.**
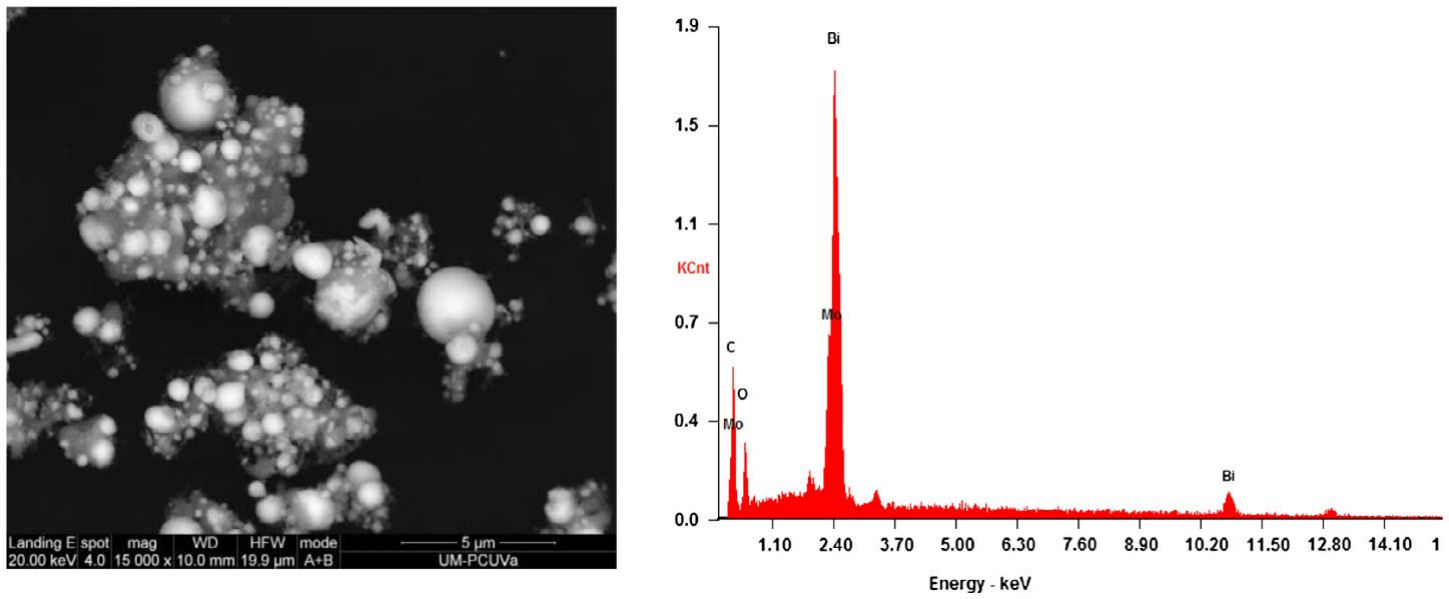
SEM micrograph (left) and EDS analysis (right) of S4 (Bi/Bi_2_MoO_6_/g-C_3_N_4_) composite.

The SEM analysis of S3 (Figure 3, left) and S4 (Figure 4, left) composites showed a large quantity of solid Bi-nanospheres in both composites, analogous to those reported by Dong et al*.* [47]. Nonetheless, in S3 the hybridization of such spheres was with rod-like Bi_2_WO_6_/g-C_3_N_4_ crystals (Figure 3, bottom), whereas in S4 a poorer crystallinity was detected for the molybdate/g-C_3_N_4_ matrix. The elemental analyses by EDS of S3 (Figure 3, right) and S4 (Figure 4, right) composites, albeit not quantitative, support these claims.

#### 3.1.3. Vibrational characterization

**Figure 5.**
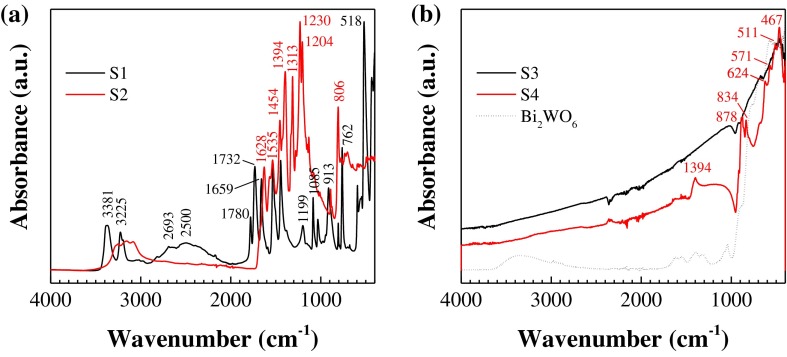
ATR-FTIR spectra of: (a) C_coke_/melamine cyanurate (S1) and C_coke_/g-C_3_N_4_ (S2) composites; (b) Bi/Bi_2_WO_6_/g-C_3_N_4_ (S3) and Bi/Bi_2_MoO_6_/g-C_3_N_4_ (S4) composites and Bi_2_WO_6_ (included for comparison purposes).

The bands in the ATR-FTIR spectrum of S1 (Figure 5(a), black line) correspond to SATS: the peaks at 3381 and 3225 cm^−1^ can be assigned to the asymmetric and symmetric NH_2_ stretching absorptions of melamine, respectively. The broad band around 2600 cm^−1^ with two peaks (2693 and 2500 cm^−1^) corresponds to amide NH interacting via hydrogen bonding with oxygen within cyanuric acid. The strong peak at 1732 cm^−1^ is allocated to NH_2_ scissoring,[55] while that at 1659 cm^−1^ relates to a NH_2_ bending vibration, as a result of the intermolecular interaction through the NH_2_ groups of the melamine molecule. The benzene ring has two intense absorption bands at 1531 cm^−1^ and 1445 cm^−1^, associated with the vibrations of C=N and C-N bonds, respectively. The position of the ν(C=O) band at 1780 cm^−1^ suggests some strengthening of the bond in the carbonyl group.[56] The peak at 1199 cm^−1^ corresponds to the bridging C-NH-C units.[57] The bands at 1085 cm^−1^ and 913 cm^−1^ originate from ring-breathing vibrations and the band at 762 cm^−1^ is due to CH wagging in the aromatic ring. The peak at 518 cm^−1^ is attributed to the side chain in-plane C–N bending vibration.[58] The main effects of the sulfuric acid treatment are the shifting of the cyanuric acid from the amide tautomer to the imidic acid one and the forming of strong hydrogen bonds between the amino groups of melamine and the oxygen of sulfate.[49]

The spectra of S2 (Figure 5(a), red line) shows the pristine g-C_3_N_4_ peaks at 1628, 1535, 1454, 1394, 1313 and 1230 cm^−1^, which correspond to the typical stretching modes of C–N heterocycles.[49] In addition, the characteristic stretching mode of triazine units at 806 cm^−1^ appears with clarity. The absence of C_coke_ bands may be attributed, on the one hand, to the small proportion of this component in the preparation of the composite and, on the other hand, to its eventual depletion (as it would act as a reductor of dehydrogenated melamine cyanurate).

FTIR spectra of S3 and S4 composites (Figure 5(b)) provide some additional evidence of the presence of g-C_3_N_4_, Bi_2_O_3_ and either Bi_2_WO_6_ or Bi_2_MoO_6_. The peak at 1394 is related to the stretching modes of CN heterocycles in g-C_3_N_4_. The peak at 878 cm^−1^ is close to the calculated *β*-C_3_N_4_ IR active mode at 891 cm^−1^, and the peak at 834 cm^−1^ can be ascribed to the characteristic breathing mode of triazine units. Nonetheless, the bands at 878 and 834 cm^−1^ may also be assigned to *ν*_1_ and *ν*_2_ of molybdate, respectively. The absorption peaks around 571 and 624 cm^−1^ correspond to the stretching vibrations of Bi-O, Mo-O and Mo-O-Mo and that 511 cm^−1^ to *ν*(Bi-O-Bi).

#### 3.1.4. Electronic and optical gap characteristics from UV–vis spectra

**Figure 6.**
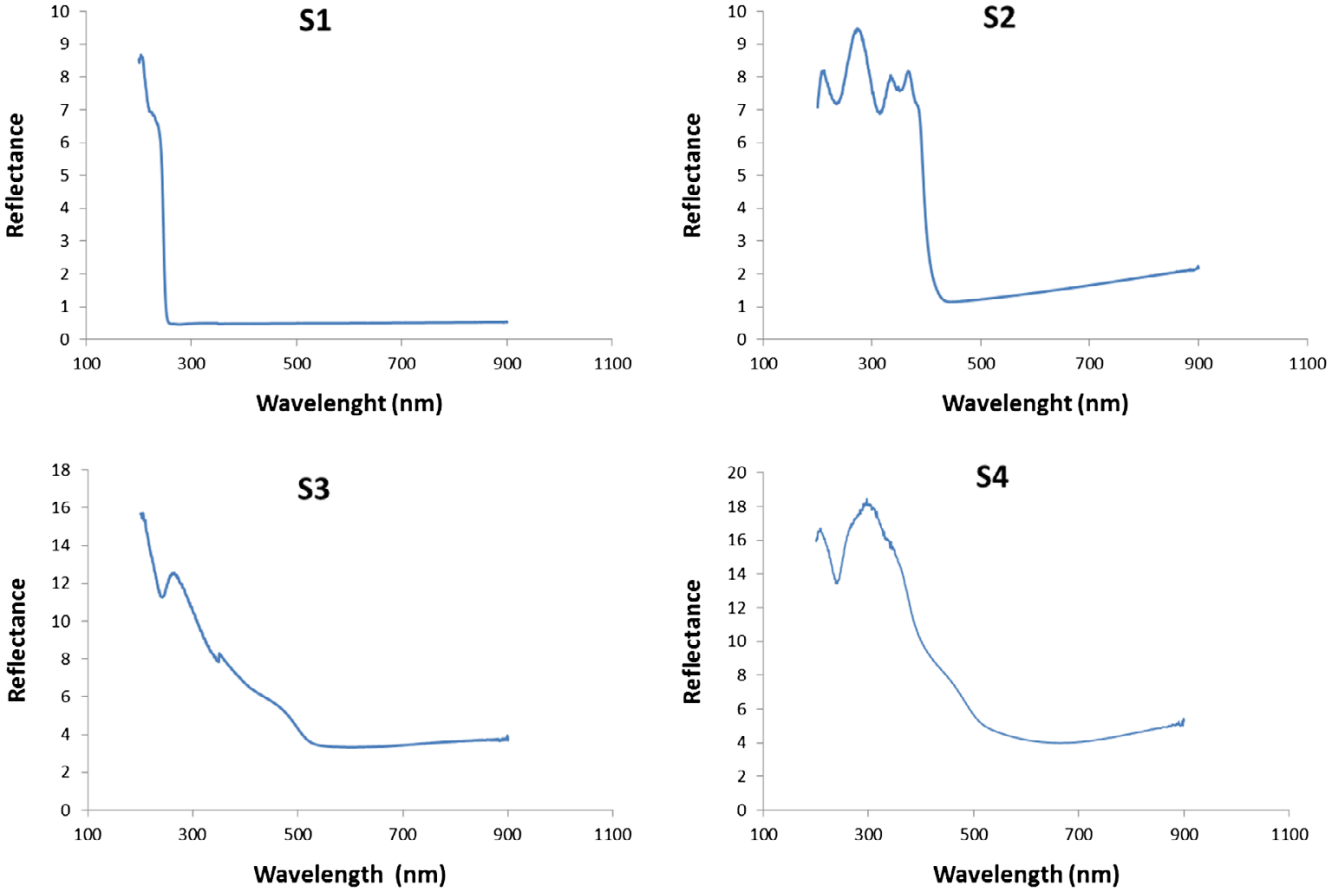
UV-vis diffuse reflectance spectra of (a) S1; (b) S2; (c) S3 and (d) S4 composites.

The UV-vis diffuse reflectance spectra of the samples are depicted in Figure 6. It can readily be observed that, whereas S1 and S2 composites only absorb in the UV region (at *λ* < 252 nm and *λ* < 410 nm respectively), S3 and S4 composites cover most of the visible spectrum, starting at *λ* < 620 nm. The estimated bandgap values *E*_*g*_ (calculated by Tauc plot method with exponent *r* = ½ [59,60]) are summarized in Table 1. While the value obtained for S2 is in the expected range for pure g-C_3_N_4_ and is typical of a wide-bandgap semiconductor, with Bi/Bi_2_MO_6_ (M = W, Mo) on g-C_3_N_4_, there is an obvious enhancement of light absorbance. It is worth noting that the *E*_g_ values of S3 and S4 composites are even smaller than that of *β*-Bi_2_O_3_ (*E*_g _= 2.50 eV) and close to those reported for WO_3_/g-C_3_N_4_ composites by Kailasam et al*.* [61]. As described in the literature,[62,63] the incorporation of Bi affects both the photocatalytic activity and the photocatalytic mechanism. Chen et al*.* [62] proposed a novel Z-scheme photocatalytic mechanism to explain the enhanced photocatalytic efficiency of Bi_2_WO_6_ and Li et al*.* [63] attributed the enhanced photocatalytic activity of Bi_2_MoO_6_ to the efficient separation of photoinduced electrons and holes. The shift of the band gap after coupling Bi_2_MO_6_ (M = W, Mo) with g-C_3_N_4_ can be attributed to the surface plasmon resonance (SPR) of Bi, because the collective excitation induced by free electrons of Bi semimetal causes strong SPR-mediated effects,[64] such as an intensive resonant visible-light absorption that can be applied in photocatalysis.

**Table 1.** Bandgap values for the composites under study.

Sample	Composition	*λ*_o_ (nm)	*E*_g_ (eV)
S1	C_coke_/melamine cyanurate	252	4.92
S2	C_coke_/g-C_3_N_4_	410	3.02
S3	Bi/Bi_2_WO_6_/g-C_3_N_4_	620	2.00
S4	Bi/Bi_2_MoO_6_/g-C_3_N_4_	620	2.00

#### 3.1.5. Photocatalytic activity

**Figure 7.**
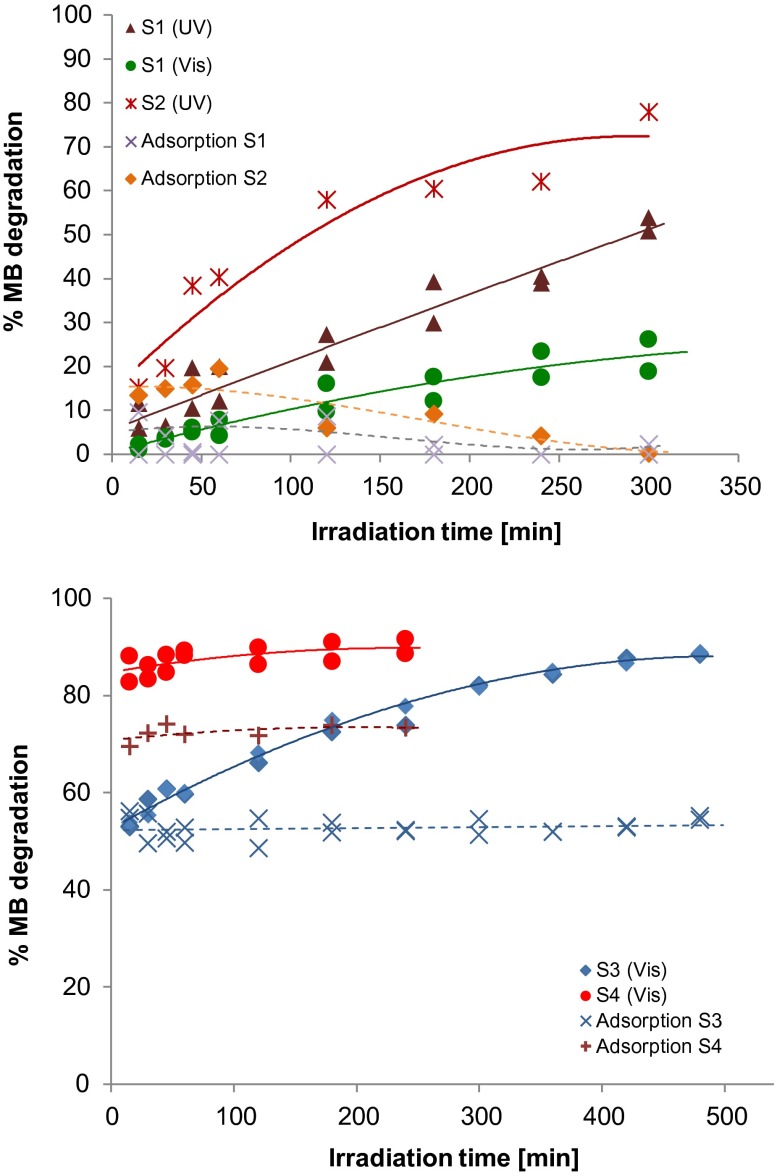
Photoactivity of the four composites under UV/visible light and in the dark.

The four composites were assessed as photocatalysts for the degradation of MB under visible irradiation (Xe lamp) and under UV light (Hg lamp). As expected from the UV-vis spectra, the photocatalytic performance of S3 and S4 under visible light was significantly better than that of the C_coke_ based composites (Figure 7). Conversely, under UV irradiation, S1 and S2 composites were more efficient than under visible irradiation. The results are in good agreement with the reported bandgap values.

From the point of view of the global decontamination of an effluent, it is also important to study the adsorption of the pollutant on the catalyst surface provided that, although it is a necessary phenomenon for the occurrence of photo-oxidation, the aim of an AOP is to completely degrade the pollutant by photo-oxidation and not to transfer the pollutant from the effluent to the solid photocatalyst, since otherwise the problem of the removal of the pollutant would not have been solved.

In the C_coke_-based samples (S1 and S2), the adsorption phenomenon (experiments conducted in the dark) can be deemed as negligible (<4%). On the other hand, for the samples with Bi/Bi_2_MO_6_ (M = W, Mo), i.e. S3 and S4, a high adsorption of the dye was detected (more than 50% within the first minute of the experiment). Consequently, the decrease in the pollutant concentration cannot be exclusively ascribed to the photocatalytic phenomenon.

## 4. Discussion

The use of a low initial C_coke_ content in the preparation of S1 and S2 has led it to behave as a reductor for polymeric carbon nitride and to the formation of C_coke_/CN_x_ composites with very low amounts of C_coke_. On the other hand, the heptazine content is only significant in S2, as a result of the thermal condensation. The heptazine content is in fact responsible for the displacement of the absorption from the UV to the visible: thus, S1, with a very low heptazine content, only absorbs at 252 nm, while S2, with an appreciable heptazine content, can absorb up to 410 nm.

Effective absorption in the visible range is only attained in S3 and S4 composites, which consist of g-C_3_N_4_, Bi metal nanospheres and Bi and W/Mo mixed oxides. The deliberate choice of a low amount of g-C_3_N_4_ in comparison to the Bi mixed oxides has caused it to act as a reductor, even leading to the formation of Bi in its metal state.

The enhanced visible-light photocatalytic performance for S3 and S4, previously reported for NO removal by a Bi/C_3_N_4_ catalyst,[47] could be due to the surface plasmon resonance endowed by Bi metal: the SPR property of Bi could conspicuously enhance visible-light-harvesting and charge separation, according to Dong et al*.* [47].

## 5. Conclusions

The C_coke_/g-C_3_N_4_ hybrid (S2), rich in heptazines, produced by thermal treatment at 550 °C of a composite made from coke and melamine cyanurate (S1), exhibited a UV-light driven photocatalytic activity but not the desirable visible-light response (with an absorption edge at 410 nm) which could allow a more efficient degradation of MB.

On the other hand, S3 and S4 composites, produced by thermal reduction of Bi_2_WO_6_ or Bi_2_MoO_6_ by g-C_3_N_4_, consisted of Bi spheres, Bi mixed oxides and g-C_3_N_4_ (Bi/Bi_2_WO_6_/g-C_3_N_4_ and Bi/Bi_2_MoO_6_/g-C_3_N_4_ composites). These nanohybrids exhibited a high and stable visible-light photocatalytic performance for the removal of MB, notwithstanding the fact that the adsorption of the contaminant could also play a role.

The use of Bi semimetal in S3 and S4 as a plasmonic cocatalyst for boosting visible light photocatalysis (in a similar fashion to Au and Pt) may provide a more economical alternative to the use of noble metals so as to advance photocatalysis efficiency.

## Disclosure statement

No potential conflict of interest was reported by the authors.

## Funding

This research was supported by the Research Project of Fundación CajaCanarias ‘FOTOCAT’ [AER01]; the Research project of Fundación CajaCanarias ‘MAGEC’ [AYE06]; and the Spanish Ministry of Economy and Competitiveness [projects ENE2013-47826-C4-1-R, ENE2013-47826-C4-4-R]. P.M.R. would like to thank Santander Universidades for its financial support through ‘@ Becas Iberoamérica Jóvenes Profesores e Investigadores, España 2016’ scholarship program.
